# Expectations Versus Reality in Inhalation Technique—A Case–Control Study of Inhalation Technique in Patients with Asthma or COPD

**DOI:** 10.3390/jcm14196848

**Published:** 2025-09-27

**Authors:** Izabela Domagała-Mańczyk, Marta Miszczuk-Cieśla, Marta Maskey-Warzęchowska, Michał Zielecki, Piotr Szczudlik, Marta Dąbrowska

**Affiliations:** 1 Department of Internal Medicine, Pulmonary Diseases and Allergy, Medical University of Warsaw, Banacha 1A, 02-097 Warsaw, Poland; izabelamdomagala@gmail.com (I.D.-M.);; 2 Department of Neurology, Medical University of Warsaw, Banacha 1A, 02-097 Warsaw, Poland

**Keywords:** chronic obstructive pulmonary disease (COPD), asthma, inhaler technique, factors associated with poor inhaler technique

## Abstract

**Background/Objectives**: Correct inhalation technique (IT) is crucial in the management of airway obstructive diseases. However, inhaler misuse among patients is frequent. The aim of the study was to assess IT and analyze factors influencing inhalation errors in adults with asthma and COPD. **Methods**: This single-center case–control study involved 180 adults with asthma or COPD. IT was evaluated using a checklist of common errors, a four-grade dedicated scale, and peak inspiratory flow. Patients with correct and incorrect IT were compared across multiple factors, including demographics, disease duration and severity, motivation for treatment, spirometry results, cognitive function, visual or hearing disorders and prior training in inhaler use. **Results**: A total of 115 patients with asthma and 65 patients with COPD were analyzed. Among them, only 59 patients (32.8%) were treated with 1 inhaler. Sixty-eight patients (37.8%) used all their inhalers properly. Correct IT was observed more frequently among DPI compared to MDI users (*p* < 0.001). Only 76 patients (42.2%) reported previous training in IT. No differences were found between correct and incorrect inhaler users (MDI or DPI) regarding age, gender, education, treatment motivation, visual or hearing impairments or cognitive disorders. Among MDI users, those with correct IT more often read the drug leaflet (*p* = 0.015). Among DPI users, proper technique was associated with better self-assessment (*p* = 0.046) and a higher rate of prior inhalation training (*p* = 0.001). **Conclusions**: Most adults with asthma or COPD do not use their inhalers properly, particularly patients using MDI. Insufficient education in the field of proper IT is still a burning issue.

## 1. Introduction

Inhaled therapy is the cornerstone in the treatment of asthma and chronic obstructive pulmonary disease (COPD). Among the wide spectrum of inhalers, the oldest are pressurized metered dose inhalers (MDI), which deliver medication as an aerosol. They most frequently contain rescue medication but may also be used in the delivery of standard therapy in both asthma and COPD. Now, the most frequently used inhalers are dry powder inhalers (DPI), and there is a wide spectrum of different inhalers in this group. Soft mist inhalers (SMI) are used relatively rarely, and their inhalation technique is similar to MDI.

Despite the wide offer of inhaler devices and various solutions to facilitate correct inhaler use, the proportion of patients with improper inhalation technique (IT) is reported to range from 25% to 80% [1,2,3,4]. Improper IT is associated with increased symptom severity and increased risk of disease exacerbations, resulting in more frequent emergency visits and hospitalizations [5,6,7]. Poor disease control, incorrect IT results in lower quality of life, worse prognosis and high healthcare resource utilization [3,4,7,8,9,10]. Moreover, inhaler misuse may lead to reduced compliance and adherence because the patient may be discouraged by the difficulties encountered during medication intake [10,11,12]. Apart from the choice of the type of medication, the selection of the optimal inhaler device should also be carefully considered and be based on the measurement of peak inspiratory flow (PIF) [10,13], assessment of the patient’s cognitive function, manual dexterity, vision acuity and patient preference.

Both the Global Initiative for Asthma (GINA) and the Global Initiative for Obstructive Lung Disease (GOLD) recommend monitoring inhalation technique on follow-up visits, particularly in patients with poor disease control, and emphasize the need to verify inhaler use in every patient who experiences a disease exacerbation [1,14,15,16]. Even as many as two-thirds (65.5%) of patients hospitalized due to COPD exacerbation make critical mistakes while using their inhalers [6]. When analyzing factors that increase the probability of worse inhalation technique, results of studies are not consistent, but some of them indicate that patients who make inhalation mistakes are more likely to be older, have cognitive impairment [17,18,19,20], more frequently use metered dose inhalers (MDI) or more than one type of inhaler and achieve lower PIF [2,4,10,13,16,21]. More frequent inhaler mishandling and lower adherence to treatment were reported for patients who did not receive instructions or an inhaler use demonstration [22,23,24]. Of note, patients who underwent IT training improved their inhaler use only temporarily [25], which supports the need for monitoring inhaler competence over time as recommended by GINA and GOLD [14,15].

The increasing awareness of the importance of competent inhaler use, clear recommendations for IT monitoring, user-friendly innovations introduced in inhaler devices, and the shift towards combined double and triple inhaled medications still have not significantly changed the incidence of inhaler misuse. In their systematic review, Sanchis et al. have shown that over the last 40 years, inhaler misuse remains at a relatively constant level of approximately 31% in patients with COPD [2]. These results point to the ongoing need to search for factors that influence improper inhaling techniques in patients with obstructive diseases.

The objective of the present study was to assess the proportion of patients with correct inhalation technique and to identify the factors influencing errors in IT in adults with asthma and COPD.

## 2. Materials and Methods

### 2.1. General Study Design

This single-center, case–control study was conducted between January 2021 and December 2023 on patients diagnosed with asthma or COPD, regularly taking inhaled medications. Inhalation technique was evaluated with the help of a checklist of the most common mistakes and rated on a 4-grade scale (1—very good, 2—good, 3—rather bad and 4—bad). IT was defined as correct when rated 1 or 2 in this 4-point scale or when a maximum of two mistakes were noted during inhalation, provided that neither of these mistakes was critical. Additionally, peak inspiratory flow (PIF) was assessed in every patient using In-Check Dial.

All patients who made any inhalation errors were offered IT training.

Patient analysis included clinical and demographic data, pulmonary function, education level, motivation for treatment, previous IT training, basic cognitive function and visual disorders.

The study was approved by the Institutional Review Board (KB/68/2019) and registered at ClinicalTrial.gov (NCT04203446). All patients signed an informed consent to participate.

### 2.2. Patients

This study included adults with asthma or COPD treated at the Department of Internal Medicine, Pulmonary Diseases and Allergy of the Medical University of Warsaw, who were admitted due to exacerbation of obstructive disease or remained under the care of the respiratory outpatient clinic. When hospitalization or a visit to the outpatient clinic was associated with obstructive disease exacerbation, IT was assessed at the end of hospitalization or during an additional follow-up visit, after the patient’s condition stabilized and the symptoms of exacerbation resolved.

The study inclusion criteria were as follows: (1) age 18–85 years; (2) diagnosis of asthma or COPD at least 3 months prior to study inclusion; and (3) recommendations of regular daily intake of inhaled medication via at least one inhaler: metered dose inhaler (MDI), dry powder inhaler (DPI) or soft mist inhaler (SMI). Exclusion criteria comprised the following: (1) seasonal or only emergency use of inhaled medications; (2) symptoms of acute respiratory infection (within 5 days of infection symptoms onset); and (3) use of MDI with a spacer.

### 2.3. Methods

The correctness of IT was assessed with the help of a checklist of the most common inhalation errors described for MDI and DPI (Supplementary Table S1) [4,16,26]. For patients who used SMI, the list of errors elaborated for MDI was applied: SMI users were grouped with MDI users for analysis. IT was further rated on a 4-grade scale, as described above. Correct IT was defined as IT rated 1 or 2, and when a maximum of two mistakes occurred during inhalation, excluding critical errors. Critical errors were defined as mistakes resulting in failure to deliver the medication dose, based on the most common mistakes reported in previous studies [4,16,27]. Additionally, peak inspiratory flow was measured by means of In-Check Dial G16 (Clement Clarke International Ltd., Wales, UK). An adequate PIF for MDI was 30–60 L/min, while for DPI– 50–120 L/min, respectively [28,29]”.

As most patients used more than one type of inhaler, factors influencing correct IT were analyzed separately for MDI (MDI grouped with SMI) and DPI users.

The following data were collected in all patients:

- General demographic and education data;
- Clinical data on asthma or COPD, as well as comorbidities;
- Data on the duration of the disease, its previous course;
- Disease control assessed with the use of the asthma control test (ACT) or COPD Assessment Test (CAT), when appropriate [30,31];
- Spirometry results (Lung Test 1000 spirometer (MES, Kraków, Poland)) [32,33];
- Disease-related quality of life (St. George’s Questionnaire for COPD and the AQLQ Quality of Life Questionnaire for Asthma, respectively) [34,35];
- Number of former IT training sessions and/or sources of information on proper IT via prescribed device;
- Motivation for treatment was evaluated with the use of a dedicated 8-item questionnaire created for the purpose of the study (Supplementary Table S2);
- Cognitive impairment assessed using the Short Mental Status Assessment Scale (MMSE) and Clock Drawing Test (CDT)—in the Polish population, with a score < 27 points suggestive of cognitive dysfunction [36,37,38];
- Potential visual or hearing impairment was evaluated in an indicative assessment using both questionnaires and ophthalmological Snellen charts.

### 2.4. Statistical Analysis

Statistical analysis was performed using the “R” programming language (Version 3, 2007; https://www.R-project.org/Licenses/)(accessed on 28 January 2025). The descriptive analysis of the survey questions was performed in the “Microsoft Excel” software. The normality of the distribution was assessed on the basis of qq charts (the “R Studio” program https://www.r-project.org/Licenses/ (accessed on 28 January 2025)). The relationships between the variables were assessed by Student’s t-test for quantitative variables with a normal distribution and the Wilcoxon test for quantitative variables with a distribution other than normal, applying the *p* value of <0.050 as the significance limit.

Based on the results of our previous studies [25], we assumed that only 20% of subjects have proper IT, and we designed the current study to find differences in the proportion of any determinants, e.g., previous inhalation training of 30% between patients who inhale properly vs. those whose IT is poor. Power analysis and sample size calculations indicated that a sample size of 155 subjects would provide 90% statistical power to detect significant differences between the two groups (alpha = 0.05, beta = 0.10).

Univariate logistic regression models were performed. Among the variables that had a significant impact on the correctness of inhalation in these models, variables were selected and included in the multivariate model of logistic regression. Collinearity of the variables was excluded. The model was evaluated by checking the AUC, AIK (Akaike’s Information Criterion) and BIC (Bayesian Information Criterion) and performing model validation. For this purpose, the research group was divided into a training group and a test group in a ratio of 7:3. The model was trained on a training set and then evaluated on a test set.

## 3. Results

A total of 180 patients were enrolled in the study—115 (63.9%) patients with asthma and 65 (36.1%) patients with COPD. An amount of 59 patients (32.8%) were treated with only 1 inhaler, 67 patients (37.2%) used 2 inhalers, while 54 patients (30%) used 3 or more inhalers. The majority of patients used at least one MDI inhaler (n = 135; 75%), 5 patients were treated with SMI (2.8%) and 110 patients used DPI (61%).

Among all patients, there were 46 subjects (25.5%) with significant visual disorders and 5 patients (2.8%) with severe hand joint or muscle problems, which might have impacted their use of inhalers. Similarly, there were 16 patients (8.9%) with significant hearing loss, which might have hindered inhalation training. Based on MMSE (performed in 153 patients), in 29 subjects (18.9%) mild cognitive impairment and in 7 patients (4.6%) mild dementia were suspected. Based on the Clock Drawing Test in two patients (1.3%), dementia might have been suspected.

Patients with COPD were significantly older than patients with asthma (*p* < 0.001) and had a shorter disease duration (*p* < 0.001), were more likely to be active or ex-smokers (*p* < 0.001) and achieved significantly lower results in lung function tests (*p* < 0.001). The detailed characteristics of the investigated group are given in Table 1, while the comparison of asthma and COPD patients is shown in Table 3.

**Figure 1:**
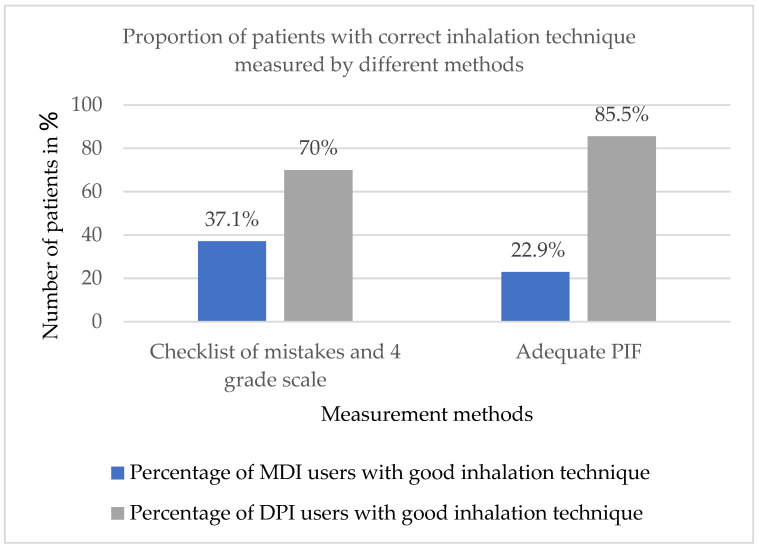
Proportion of patients with good inhalation technique.

In the whole group, 68 patients (37.8%) used all their inhalers properly. The proportion of patients with very good or good IT was higher in DPI users than in MDI users (77/110 (70%) for DPI vs. 52/140 (37.1%) for MDI, *p* < 0.001) (Figure 1). Among patients using MDI, the median PIF with appropriate simulated resistance was 110 l/min (IQR 70–120), and, among patients taking DPI, it was 60 L/min (42.5–70). A PIF value required for proper inhaler use was documented in 94/110 (85.5%) in subjects using DPI and 32/140 (22.9%) in MDI users (*p* < 0.001) (Figure 1).

**Figure 2:**
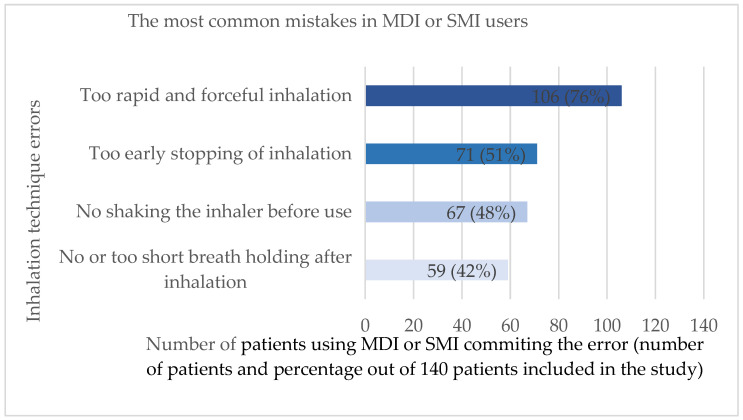
The most common mistakes made by patients using metered dose (MDI) or soft mist inhalers (SMI) (N = 140). Analysis based on 140 patients committing multiple errors.

**Figure 3:**
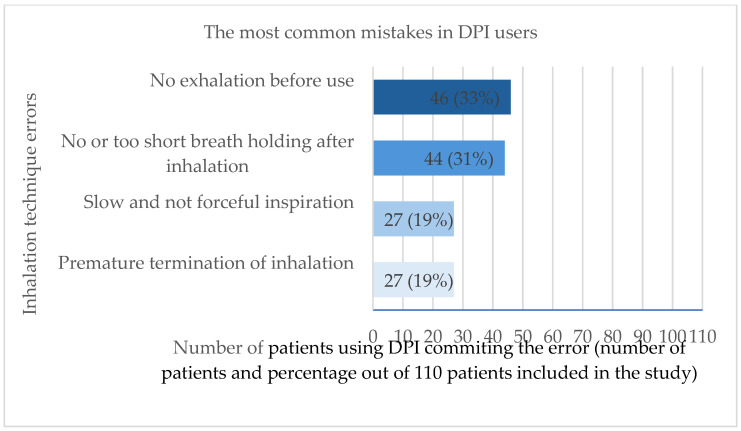
The most common mistakes made by patients using dry powder inhalers (DPI) (N = 110). Analysis based on 110 patients committing multiple errors.

The most common mistakes in MDI and DPI users are shown in Figure 2 and Figure 3, respectively.

**Figure 4:**
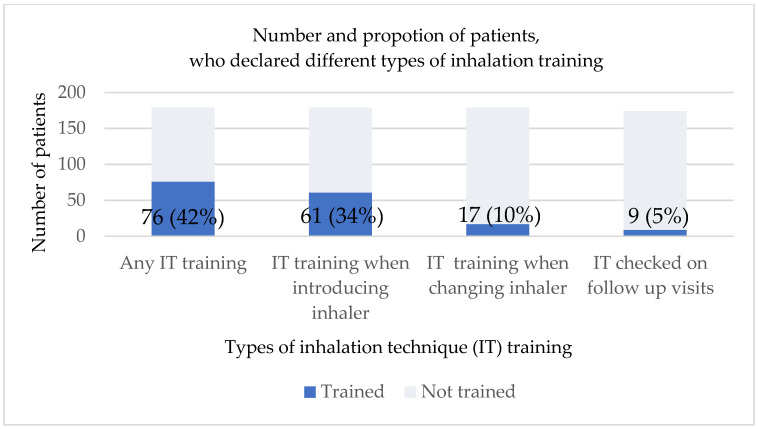
Number of patients who declared inhalation training.

In the pool, only 76 patients (42.2%) reported they had previously undergone any IT training. At the start of treatment, 61 patients (33.9%) were instructed how to use their new inhaler. Seventeen patients (9.4%) received IT training during treatment or when changing to a new inhaler, while nine (5%) patients declared that IT was checked at each visit (Figure 4).

No differences between patients who correctly and incorrectly used inhaled medication (both MDI and DPI) were identified in terms of age, gender, education level, motivation for treatment, coexistence of significant visual or hearing impairment, cognitive disorders or problems with hand joints that could potentially hinder the proper administration of inhaled drugs (Table 2).

Among MDI users, subjects using MDI correctly read the drug leaflet more frequently than patients with incorrect IT (*p* = 0.015), but no difference was found for the frequency of previous inhalation training between these two groups. Patients who demonstrated correct technique for MDI declared more drug-related side effects (*p* = 0.004).

Patients who used DPI correctly had a better self-assessment of their IT (*p* = 0.046) and were more frequently trained how to use inhalers than patients who used DPI incorrectly (*p* = 0.001) (Table 2).

Patients with COPD significantly less often received IT training when starting treatment or when switching to a new inhaled drug when compared to patients with asthma (*p* = 0.012 and *p* = 0.003, respectively). However, we did not find differences in the proportion of patients with correct and incorrect inhaler use between subjects with asthma or COPD (Table 3).

Univariate analysis revealed several factors that had a significant impact on proper inhalation technique (Supplementary Table S3). Further, multivariate logistic regression showed that treatment with MDI and deviations from regular use of inhalers promote poor IT, while higher self-assessment of good IT was related to correct inhaler use. The sensitivity of this logistic regression model was 0.66 (95% CI: 0.48–0.77), the specificity was 0.66 (95% CI: 0.50–0.78) and the internal accuracy AUC was 0.69 (95% CI: 0.61–0.77) (Table 4).

## 4. Discussion

The results of the study indicate that a significant proportion of patients with asthma or COPD still use more than one type of inhaler, and only more than one-third of them can use their inhalers correctly. Next, improper IT is more common in patients using MDI and those who use inhalers irregularly, while age, gender, duration and the course of obstructive disorder and the coexistence of pattern disorders, hearing disorders, joint diseases or cognitive disorders are not associated with a higher risk of inhaler incompetence. Insufficient education of patients in the field of correct IT still seems to be a burning issue.

A low percentage of patients who correctly take inhaled medication was observed by many other authors, e.g., in the studies by Bao et al. (18.7%) [39] or Melzer et al. (34.5%) [40] and in our previous studies (20%) [25]. In this study, 37.8% of patients used all their inhalers properly, but this proportion differed significantly between MDI and DPI users. Interestingly, such disproportion was also observed for PIF measurements with predominance of adequate PIF in DPI users. This suggests that slow and steady inspiration is difficult for a substantial proportion of patients and points to a need for thorough and repeated instruction on inhalation speed, especially in MDI users. As a matter of fact, the range of adequate PIF for MDI has recently been reassessed since new studies documented that in new MDI, including HFA as propellant, a maximum flow rate of 120 L/min is defined as an upper limit for optimal PIF [41]. However, in our study, the optimal range of PIF 30–60 L/min was used based on earlier studies and statements [28,29], as most devices on the market are generic and product-specific data, including the range of optimal PIF, are not available.

Despite progress in improving inhalers and increasing interest in environmentally friendly inhalers [42,43,44], real-life research shows that we are not yet able to effectively improve IT. In this context, the identification of groups of patients who are at risk of improper inhaler use is necessary. Our study reveals that patients using MDI are more likely to make errors in IT than DPI users, which is consistent with the results of previous studies [45,46,47,48]. This indicates the need to focus on patients who inhale their medication via MDI. Based on the literature, if there is a persistent problem with proper IT via MDI, the solution is the use of MDI along with an inhalation chamber [49,50].

Additionally, both the GINA and GOLD and numerous studies [1,14,21,29] pointed to a higher risk of incorrect IT in patients using several types of inhalers. In our study, 67.2% of patients were still treated with two or more inhalers, undoubtedly contributing to the high percentage of patients whose IT was not optimal.

Surprisingly, we did not identify differences between patients who used inhaled medications correctly and incorrectly in terms of age, gender, education level, motivation for treatment, visual or hearing disorders, cognitive disorders or problems with dexterity due to hand disability. However, we observed that better self-assessment of IT and fewer deviations in inhaler use were associated with correct technique, which results in better adherence to treatment, reduces symptom intensity and improves patient quality of life [51]. Previous studies have also demonstrated that it increases patient satisfaction and motivation for treatment [10,52].

While previous studies [4,18,39,47,53,54,55,56] show that older people are more likely to make mistakes in IT, we found no significant effect of age on proper IT in our study. Some researchers suggest that a higher number of mistakes made in the elderly may be related to cognitive impairment [18,47], but patients with mild cognitive impairment can still take inhaled medications properly [53]. Most studies on IT exclude patients with severe cognitive impairment. Although severe cognitive impairment was not an exclusion criterion in our study, only seven subjects were preliminarily diagnosed with mild dementia based on MMSE assessment, and, in 2 patients, dementia was suspected based on neurological assessment of CDT. We may assume that due to the small number of people with cognitive impairments in our study, we did not find a relationship between the occurrence of neurological disorders and the number of errors made in IT. We observed a higher incidence of cognitive impairment in the group of patients who took their inhaled medication incorrectly (*p* = 0.090). This only indicates that this group should be frequently checked and trained in the scope of IT.

Another factor that may affect IT in elderly patients is degenerative joint disease [39,55]. According to Usmani et al. [21], in the elderly, common challenges in proper inhalation include difficulty manipulating the device due to dexterity, osteoarthritis or hand joint pain, which may hinder inhaled therapy. In our study, osteoarthritis was diagnosed in 27 subjects, but only 5 of them declared dexterity or problems with using inhalers due to a hand disability. Such a low number of subjects might have been insufficient to identify a relationship between dexterity and improper IT.

Our study included adults with a median age of 63.5 years, but inhalation mistakes are also common in younger groups [57,58,59]. In the study by Almomani et al., including children and adolescents with asthma aged 7 to 17 years, more than half of the patients used more than one type of inhaler, and more mistakes were noted in MDI users. Moreover, worse IT was associated with a lower level of parental education [58,60]. Correct IT and good adherence were found to be positively associated with disease control, as well as in children [60]. It has been documented that repeated training of proper IT is of great importance in all patients, independent of their age [57]. The results of our study did not demonstrate the predisposition of any gender to inferior IT. Previous studies showed that women may be more susceptible to inhaler incompetence [54,61,62], but other studies demonstrated that errors in inhaling were similarly common in both sexes [46,56,63,64], or more errors were made by men [47].

Selected previous studies indicate that lower educational attainment was associated with increased odds of poor IT for nearly all devices [4,40], but in our study, we did not observe differences in the frequency of incorrect inhalation of medications depending on education.

A key modifiable factor influencing IT is education and training, which has been demonstrated in many previous studies [2,4,25,65]. There is an increasing number of studies on different methods of inhalation training [65,66,67,68,69]. All of the above studies prove that IT training leads only to a temporary IT improvement; it has not been determined which type of training is the most effective, and, by consequence, no standard method of IT training has been established so far [11]. In the recently published review and meta-analysis by Marko and Pawliczak, the authors did not identify an educational approach resulting in improved patients’ inhalation skills [11].

According to previous research, training in IT should be repeated at least every 3 months in each patient [25,65]. Yet, there is a high discrepancy between the recommendations and everyday practice. In our study, only 42.2% of patients had been previously trained, of which only 5.2% declared that their IT was checked at each medical visit. It clearly points to a high discrepancy between recommendations and real-life practice. We cannot exclude that such an assessment may be burdened with the recall bias, but similar results were published by Bao et al., who found that only 41.8% of elderly patients with COPD were instructed in IT [39]. In our study, we observed that patients with COPD were significantly less likely to be trained in IT compared to patients with asthma. When inhaler type was considered, patients using DPI correctly were significantly more often trained compared to patients taking DPI incorrectly, and patients using MDI correctly were significantly more likely than patients incorrectly using MDI to read drug leaflets before and during treatment.

Several limitations should be considered in the analysis of our study. First, it was a single-center study with a limited number of patients. Second, IT was evaluated by one investigator only; however, this was a respiratory physician with experience in IT evaluation and training, and the agreement of this investigator’s assessment with that of other investigators has been documented in a different study (Miszczuk-Ciesla et al., in press). The application of a dedicated four-grade scale for IT evaluation may raise doubts about some subjectiveness in the assessment, but similar scales (three- or five-grade) were used in some earlier studies [70,71]. Next, the preliminary results of the comparison of this scale with the checklist of inhalation mistakes suggest that the four-grade scale was more reliable than the checklist of mistakes [72]. To the best of our knowledge, there is no validated scale for IT evaluation to date. Moreover, we also applied the list of the most common inhaler mistakes described in the literature to increase the credibility of our rating. Such a mistake checklist is the most popular method of IT assessment, but with the development of new inhalers, the perception of inhalation errors is evolving. A good example would be the issue of shaking MDI before use. With the introduction of new propellants in MDI, shaking the inhaler became a negligible maneuver, but such information is not clearly visible in many drug leaflets, which may be misleading for patients. Thus, in this study, we decided to include the lack of inhaler shaking before use as a mistake; however, it was not labeled as a critical one.

Finally, visual, hearing or mental disorders were relatively rare in our investigated population, which might have had an impact on related results. On the other hand, the upper age limit for enrolment in the study was 85 years, to exclude selection bias. Perhaps, with the aging of the population, there is a need for studies in even older age groups and studies focused on patients with such disabilities.

## 5. Conclusions

The results of this real-life study confirm that the majority of adults with asthma or COPD do not use their inhalers properly, particularly patients using MDI or those who tend to use inhalers irregularly. Insufficient education in the field of proper inhalation technique seems to be a major obstacle in the improvement of inhalation technique in patients with obstructive lung diseases.

## Figures and Tables

**Table 1:** Patients’ characteristics.

	The Whole Group	Asthma	COPD
Number of patients	180	115	65
Age (years)	63.5 (52–71)	60 (44–66.5)	70 (64–76)
Sex F/M	104/76	76 (66.1%)/39 (33.9%)	28 (43.1%)/37 (56.9%)
Duration of the disease (years)	10 (6–20)	15 (7–26.5)	10 (5–15)
Number of used inhalers	2 (1–2)	2 (1–2)	2 (2–2)
Smoking history (NS/S/EX)	61/15/104	58/7/50	3/8/54
FEV_1_% N	69% (45–88)	81% (61–95.5)	44.5% (37–65)
Education (primary/secondary/higher)	13/95/72	6/59/50	7/36/22
Number of comorbidities	1 (1–3)	1 (1–2)	2 (0–3)
Asthma Control Test (points)	X	20 (16–23.5)	X
Quality of Life—AQLQ (points)	X	5.48 (4.29–6.23)	X
COPD Assessment Test (points)	X	X	22 (16–26)
Quality of Life—SGRQ (points)	X	X	56.9 (35.9–70)

Data are presented as median and IQR or numbers of patients. Abbreviations: AQLQ, Asthma Quality of Life Questionnaire; EX, ex-smoker; F, female; FEV_1_%N, forced expiratory volume in 1st second expressed as percentage of predicted value; M, male; NS, never smoker; SGRQ, Saint George’s Respiratory Questionnaire; S, smoker.

**Table 2:** Comparison of patients with proper and incorrect inhalation technique.

	MDI/SMI N = 140			DPI N = 110		
	Proper Inhalation Technique N = 52	Incorrect Inhalation Technique N = 88	*p* Value	Proper Inhalation Technique N = 77	Incorrect Inhalation Technique N = 33	*p* Value
Age (years)	62.5 (51.8–70)	65.5 (55.8–71)	0.267	64 (55–71)	63 (54–70)	0.854
Asthma/COPD	35 (67.3%)/17 (32.7%)	56 (63.6%)/ 32 (26.4%)	0.797	45 (58.4%)/ 32 (41.6%)	18 (54.5%)/ 15 (45.4%)	0.866
Disease duration (years)	10 (6–22)	11 (6,8–20)	0.761	10 (7–20)	10 (5–20)	0.786
Gender (F/M)	32 (61.5%)/ 20 (38.5%)	48 (54.5%)/ 40 (45.5%)	0.528	40 (51.9%)/37 (48.1%)	20 (60.6%)/13 (39.4%)	0.531
Smoking history (S/ExS, NS)	3/38/11	10/47/31	0.103	7/43/27	4/18/11	0.887
Education (primary/ secondary/ higher)	5 (9.6%)/ 23 (46.2%)/ 24 (46.2%)	6 (6.8%)/ 55 (62.5%)/ 27 (30.7%)	0.109	4 (5.2%)/ 43 (55.8%)/ 30 (38.9%)	4 (12.1%)/ 19 (57.6%)/ 10 (30.3%)	0.366
Motivation for therapy (points)	10 (9–10)	10 (9–10)	0.484	10 (8–10)	10 (9–10)	0.640
Number of inhalers	2 (1–2)	2 (2–2)	0.063	2 (2–2)	2 (1.8–2)	0.753
FEV1%N z-score	−1.6 (−2.4; −0.5)	−2.4 (−2.4; −1.2)	0.109	−1.9 (−2.4; −1.3)	−2.4 (−2.4; −1.1)	0.534
Side effects of the inhalers	1 (1–3)	1 (0–2)	0.004	1 (0–2)	1 (0–2)	0.534
Significant visual disorders	12 (37.5%)	33 (29.7%)	0.897	24 (31.2%)	11 (33.3%)	0.823
Significant hearing loss	4 (7.7%)	8 (9.1%)	0.775	10 (13%)	0	0.089
Hand muscle/joint disorders	0	4 (4.5%)	0.408	3 (18.8%)	1 (33.3%)	0.597
Any cognitive disorders (MMSE)	6 (11.5%)	20 (22.7%)	0.099	14 (20.3%)	10 (35.7%)	0.158
Regular use of inhalers in self-assessment ^1^	51 (98.1%)	84 (95.4%)	0.736	73 (94.8%)	31 (93.9%)	0.854
Any deviations from regular use of inhalers during last 6 months ^1^	6 (11.5%)	21 (23.9%)	0.118	11 (14.3%)	10 (30.3%)	0.090
Positive self-assessment of own inhalation technique ^1^	50 (96.2%)	80 (90.9%)	0.410	76 (98.7%)	29 (87.9%)	0.046
Any IT before	23 (45.1%)	36 (40.9%)	0.761	38 (49.4%)	4 (12.1%)	0.001
IT when introducing treatment	12 (23.5%)	30 (34.1%)	0.265	32 (41.6%)	3 (9.1%)	0.002
IT when changing Inhaler	4 (7.8%)	8 (9.1%)	0.775	11 (14.3%)	0	0.052
Reading the drug leaflet	49 (96.1%)	70 (79.6%)	0.015	65 (84.4%)	28 (84.9%)	0.954

Data are given as median and interquartile range or number and percentages. Abbreviations: ExS, Ex-smoker; F, female; FEV_1_%N, forced expiratory volume in 1st second expressed as z-score; IT, inhalation training; M, male; S, smoker; NS, never smoker. ^1^ Questionnaire assessing motivation for treatment (Supplementary Table S2).

**Table 3:** Comparison of patients with asthma and COPD.

	Asthma N = 115	COPD N = 65	*p* Value
Age	60 (44–66.5)	70 (64–76)	<0.001
Disease duration	15 (7–26.5)	10 (5–15)	<0.001
Motivation	10 (8–10)	10 (9–10)	0.443
Number of inhalers	2 (1–2)	2 (2–2)	0.652
Gender (F/M)	76 (66.1%)/39 (33.9%)	28 (43.1%)/37 (56.9%)	0.004
Number of comorbidities	1 (1–2)	2 (0–3)	0.264
Any inhalation training before	61 (53.5%)	15 (23.1%)	<0.001
Any inhalation training when starting treatment	47 (41.2%)	14 (21.5%)	0.012
Any inhalation training when switching the inhaler	17 (14.9%)	0	0.003
Reading the drug leaflet	100 (87.7%)	55 (84.6%)	0.720
Number of side effects related to inhalers	1 (0–2)	1 (0–2)	0.228
Proper inhalation technique for DPI	26 (41.3%)	21 (44.7%)	0.913
Proper inhalation technique for MDI	11 (12.1%)	5 (10.2%)	0.951
Proper peak inspiratory flow by In-Check Dial in DPI users	53 (86.9%)	41 (89.1%)	0.958
Proper peak inspiratory flow by In-Check Dial in MDI users	21 (22.6%)	11 (22.0%)	0.937

**Table 4:** Multivariate logistic regression analysis of factors related to proper inhalation technique.

Factor	OR	SE	*p* Value	95%CI
Good self-assessment of inhalation technique	1.50	0.15	0.08	1.12–2.02
Deviations from regular use of inhaler during last 6 months	0.83	0.09	0.03	0.70–0.98
Use of MDI	0.79	0.09	0.01	0.66–0.94
Use of both types of inhalers	1.00	0.01	0.99	0.84–1.20

## Data Availability

The datasets analyzed during the current study contain sensitive patient information and are therefore not publicly available. Anonymized data are available from the corresponding author on request and subject to institutional and ethical approvals.

## Supplementary Materials

The following supporting information can be downloaded at: https://www.mdpi.com/article/10.3390/jcm14196848/s1; Table S1: checklist of the potential mistakes during inhalation; Table S2: questionnaire assessing motivation for treatment. Table S3: Factors that impact on proper inhalation technique- univariate analysis.

## Abbreviations

The following abbreviations are used in this manuscript:

MDI	Metered Dose Inhaler
DPI	Dry Powder Inhaler
COPD	Chronic Obstructive Pulmonary Disease
IT	Inhalation Technique
PIF	Peak Inspiratory Flow

## References

[1] Skolnik N., Yawn B.P., de Sousa J.C., Vázquez M.M.M., Barnard A., Wright W.L., Ulrich A., Winders T., Brunton S. (2024). Best practice advice for asthma exacerbation prevention and management in primary care: An international expert consensus. npj Prim. Care Respir. Med..

[2] Sanchis J., Gich I., Pedersen S. (2016). Systematic Review of Errors in Inhaler Use: Has Patient Technique Improved Over Time?. Chest.

[3] Molimard M., Raherison C., Lignot S., Balestra A., Lamarque S., Chartier A., Droz-Perroteau C., Lassalle R., Moore N., Girodet P.-O. (2017). Chronic obstructive pulmonary disease exacerbation and inhaler device handling: Real-life assessment of 2935 patients. Eur. Respir. J..

[4] Usmani O.S., Lavorini F., Marshall J., Dunlop W.C.N., Heron L., Farrington E., Dekhuijzen R. (2018). Critical inhaler errors in asthma and COPD: A systematic review of impact on health outcomes. Respir. Res..

[5] Melani A.S., Bonavia M., Cilenti V., Cinti C., Lodi M., Martucci P., Serra M., Scichilone N., Sestini P., Aliani M. (2011). Inhaler mishandling remains common in real life and is associated with reduced disease control. Respir. Med..

[6] Grandmaison G., Grobéty T., Vaucher J., Hayoz D., Suter P. (2024). Prevalence of Critical Errors and Insufficient Peak Inspiratory Flow in Patients Hospitalized with COPD in a Department of General Internal Medicine: A Cross-Sectional Study. Chronic Obstr. Pulm. Dis..

[7] Halpin D.M.G., Mahler D.A. (2024). Systematic review of the effects of patient errors using inhaled delivery systems on clinical outcomes in COPD. BMJ Open Respir. Res..

[8] Leving M.T., van Boven J.F.M., Bosnic-Anticevich S.Z., van Cooten J., de Sousa J.C., Cvetkovski B., Dekhuijzen R., Dijk L., Pardo M.G., Gardev A. (2022). Suboptimal Peak Inspiratory Flow and Critical Inhalation Errors are Associated with Higher COPD-Related Healthcare Costs. Int. J. Chronic Obstruct. Pulmon. Dis..

[9] Kocks J., Bosnic-Anticevich S., van Cooten J., de Sousa J.C., Cvetkovski B., Dekhuijzen R., Dijk L., Pardo M.G., Gardev A., Gawlik R. (2023). Identifying critical inhalation technique errors in Dry Powder Inhaler use in patients with COPD based on the association with health status and exacerbations: Findings from the multi-country cross-sectional observational PIFotal study. BMC Pulm. Med..

[10] Calle Rubio M., Adami Teppa P.J., Rodríguez Hermosa J.L., García Carro M., Tallón Martínez J.C., Riesco Rubio C., Fernández Cortés L., Morales Dueñas M., Chamorro del Barrio V., Sánchez-del Hoyo R. (2025). Insights from Real-World Evidence on the Use of Inhalers in Clinical Practice. J. Clin. Med..

[11] Marko M., Pawliczak R. (2025). Inhalation technique-related errors after education among asthma and COPD patients using different types of inhalers—Systematic review and meta-analysis. npj Prim. Care Respir. Med..

[12] Marko M., Klimczak M., Sobczak M., Wojakiewicz M., Dębowski T., Emeryk A., Pawliczak R. (2025). Effective inhaler technique education is achievable—Assessment and comparison of five inhaler devices errors. Front. Pharmacol..

[13] Pankovitch S., Frohlich M., AlOthman B., Marciniuk J., Bernier J., Paul-Emile D., Bourbeau J., Ross B.A. (2025). Peak Inspiratory Flow and Inhaler Prescription Strategies in a Specialized COPD Clinical Program: A Real-World Observational Study. Chest.

[14] Global Initiative for Asthma (GINA) Global Strategy for Asthma Management and Prevention. 2024 Report. https://ginasthma.org/2024-report/.

[15] Global Initiative for Chronic Obstructive Lung Disease (GOLD) Global Strategy for Prevention, Diagnosis and Management of COPD. 2025 Report. https://goldcopd.org/2025-gold-report/.

[16] Kocks J.W.H., Chrystyn H., van der Palen J., Thomas M., Yates L., Landis S.H., Driessen M.T., Gokhale M., Sharma R., Molimard M. (2018). Systematic review of association between critical errors in inhalation and health outcomes in asthma and COPD. npj Prim. Care Respir. Med..

[17] Bohadana A., Jarjoui A., Lujan R., Jaffal S., Rokach A., Izbicki G. (2025). Inhaler Technique, Critical Errors, and Effective Inspiratory Flow in COPD Patients: A Prospective Study Comparing Patients Over and Under 65 Years of Age. J. Aerosol Med. Pulm. Drug Deliv..

[18] Allen S.C., Ragab S. (2002). Ability to learn inhaler technique in relation to cognitive scores and tests of praxis in old age. Postgrad. Med. J..

[19] Song M.J., Kim S.Y., Kang Y.A., Kim Y.S., Park M.S., Ye B.S., Jung J.Y. (2022). The relationship between cognitive function and competence in inhaler technique in older adults with airway disease. Geriatr. Nurs..

[20] Elander A., Gustafsson M. (2020). Inhaler Technique and Self-reported Adherence to Medications Among Hospitalised People with Asthma and COPD. Drugs Real World Outcomes.

[21] Usmani O.S. (2019). Choosing the right inhaler for your asthma or COPD patient. Ther. Clin. Risk Manag..

[22] Matsuyama T., Machida K., Hamu A., Takagi K., Momi H., Higashimoto I., Inoue H. (2022). Effects of instructional materials on the proper techniques of inhaler device use. Respir. Investig..

[23] Marando M., Tamburello A., Diedrich J.P., Valenti A., Gianella P. (2022). Effectiveness of an Educational Intervention on Inhaler Technique Proficiency in Chronic Obstructive Pulmonary Disease: A Single-Center Quality Improvement Study. J. Respir..

[24] Basheti I.A., Obeidat N.M., Reddel H.K. (2017). Effect of novel inhaler technique reminder labels on the retention of inhaler technique skills in asthma: A single-blind randomized controlled trial. npj Prim. Care Respir. Med..

[25] Dabrowska M., Luczak-Wozniak K., Miszczuk M., Domagala I., Lubanski W., Leszczynski A., Maskey-Warzechowska M., Rubinsztajn R., Hermanowicz-Salamon J., Krenke R. (2019). Impact of a Single Session of Inhalation Technique Training on Inhalation Skills and the Course of Asthma and COPD. Respir. Care.

[26] Bosnic-Anticevich S., Bender B.G., Shuler M.T., Hess M., Kocks J.W. (2023). Recognizing and Tackling Inhaler Technique Decay in Asthma and Chronic Obstructive Pulmonary Disesase (COPD) Clinical Practice. J. Allergy Clin. Immunol. Pract..

[27] Price D.B., Román-Rodríguez M., McQueen R.B., Bosnic-Anticevich S., Carter V., Gruffydd-Jones K., Haughney J., Henrichsen S., Hutton C., Infantino A. (2017). Inhaler Errors in the CRITIKAL Study: Type, Frequency, and Association with Asthma Outcomes. J. Allergy Clin. Immunol. Pract..

[28] Chrystyn H., Price D. (2009). Not all asthma inhalers are the same: Factors to consider when prescribing an inhaler. Prim. Care Respir. J..

[29] Laube B.L., Janssens H.M., de Jongh F.H., Devadason S.G., Dhand R., Diot P., Everard M.L., Horvath I., Navalesi P., Voshaar T. (2011). What the pulmonary specialist should know about the new inhalation therapies. Eur. Respir. J..

[30] Gupta N., Pinto L.M., Morogan A., Bourbeau J. (2014). The COPD assessment test: A systematic review. Eur. Respir. J..

[31] Nathan R.A., Sorkness C.A., Kosinski M., Schatz M., Li J.T., Marcus P., Murray J.J., Pendergraft T.B. (2004). Development of the asthma control test: A survey for assessing asthma control. J. Allergy Clin. Immunol..

[32] Quanjer P.H., Stanojevic S., Cole T.J., Baur X., Hall G.L., Culver B.H., Enright P.L., Hankinson J.L., Ip M.S.M., Zheng J. (2012). Multi-ethnic reference values for spirometry for the 3–95-yr age range: The global lung function 2012 equations. Eur. Respir. J..

[33] Stanojevic S., Kaminsky D.A., Miller M.R., Thompson B., Aliverti A., Barjaktarevic I., Cooper B.G., Culver B., Derom E., Hall G.L. (2022). ERS/ATS technical standard on interpretive strategies for routine lung function tests. Eur. Respir. J..

[34] Juniper E.F., Buist A.S., Cox F.M., Ferrie P.J., King D.R. (1999). Validation of a standardized version of the Asthma Quality of Life Questionnaire. Chest.

[35] Jones P.W., Quirk F.H., Baveystock C.M., Littlejohns P. (1992). A self-complete measure of health status for chronic airflow limitation: The St. George’s Respiratory Questionnaire. Am. Rev. Respir. Dis..

[36] Folstein M.F., Folstein S.E., McHugh P.R. (1975). “Mini-mental state”. A practical method for grading the cognitive state of patients for the clinician. J. Psychiatr. Res..

[37] Pracownia Testów Psychologicznych PTP (2025). MMSE—Mini Mental State Examination: Polska Adaptacja i Normalizacja Testu. Podręcznik Użytkownika.

[38] Mossakowska M., Więcek A., Błędowski P. (2020). Aspekty Medyczne, Psychologiczne, Socjologiczne i Ekonomiczne Starzenia się Ludzi w Polsce.

[39] Bao L.K., Khoa N.D., Chi L.T.K., Anh N.T. (2023). Prevalence and Factors Affecting Appropriate Inhaler Use in Elderly Patients with Chronic Obstructive Pulmonary Disease: A Prospective Study. J. Clin. Med..

[40] Melzer A.C., Ghassemieh B.J., Gillespie S.E., Lindenauer P.K., McBurnie M.A., Mularski R.A., Naureckas E.T., Vollmer W.M., Au D.H. (2017). Patient characteristics associated with poor inhaler technique among a cohort of patients with COPD. Respir. Med..

[41] Mahler D.A. (2020). The role of inspiratory flow in selection and use of inhaled therapy for patients with chronic obstructive pulmonary disease. Respir. Med..

[42] Usmani O.S., Levy M.L. (2023). Effective respiratory management of asthma and COPD and the environmental impacts of inhalers. npj Prim. Care Respir. Med..

[43] Janson C., Henderson R., Löfdahl M., Hedberg M., Sharma R., Wilkinson A.J.K. (2020). Carbon footprint impact of the choice of inhalers for asthma and COPD. Thorax.

[44] Vartiainen V., Woodcock A.A., Wilkinson A., Janson C., Björnsdóttir U., Haahtela T., Lehtimäki L. (2024). Thoughtful prescription of inhaled medication has the potential to reduce inhaler-related greenhouse gas emissions by 85. BMJ Open Respir. Res..

[45] Molimard M., Raherison C., Lignot S., Depont F., Abouelfath A., Moore N. (2003). Assessment of handling of inhaler devices in real life: An observational study in 3811 patients in primary care. J. Aerosol Med..

[46] Alotaibi M.M., Hughes L., Ford W.R. (2023). Assessing Inhaler Techniques of Asthma Patients Using Aerosol Inhalation Monitors (AIM): A Cross-Sectional Study. Healthcare.

[47] Iamthanaporn C., Wisitsartkul A., Chuaychoo B. (2023). Cognitive impairment according to Montreal Cognitive Assessment independently predicts the ability of chronic obstructive pulmonary disease patients to maintain proper inhaler technique. BMC Pulm. Med..

[48] Zhou L.-J., Wen X.-X., Jiang R., Zhou H.-Y., Li Y., Mao X.-R., Lan M. (2022). Inhaler use in chronic obstructive pulmonary disease patients: A meta-analysis. Front. Nurs..

[49] Nicola M., Soliman Y.M.A., Hussein R., Saeed H., Abdelrahim M. (2020). Comparison between traditional and nontraditional add-on devices used with pressurised metered-dose inhalers. ERJ Open Res..

[50] Abdelfattah A.M., Sarhan R.M., Madney Y.M., Mady A.F., Abdelrahim M.E.A., Harb H.S. (2024). User-Friendliness Evaluation of Handling pMDI with Various Add-on Devices in Asthmatic Patients. AAPS PharmSciTech.

[51] Göriş S., Taşci S., Elmali F. (2013). The effects of training on inhaler technique and quality of life in patients with COPD. J. Aerosol Med. Pulm. Drug Deliv..

[52] Cushen B., Sulaiman I., Greene G., MacHale E., Mokoka M., Reilly R.B., Bennett K., Doyle F., van Boven J.F.M., Costello R.W. (2018). The Clinical Impact of Different Adherence Behaviors in Patients with Severe Chronic Obstructive Pulmonary Disease. Am. J. Respir. Crit. Care Med..

[53] Maricoto T., Santos D., Carvalho C., Teles I., Correia-de-Sousa J., Taborda-Barata L. (2020). Assessment of Poor Inhaler Technique in Older Patients with Asthma or COPD: A Predictive Tool for Clinical Risk and Inhaler Performance. Drugs Aging.

[54] Chorão P., Pereira A.M., Fonseca J.A. (2014). Inhaler devices in asthma and COPD—An assessment of inhaler technique and patient preferences. Respir. Med..

[55] Usami O. (2022). Improved inhaler handling after repeated inhalation guidance for elderly patients with bronchial asthma and chronic obstructive pulmonary disease. Medicine.

[56] Schreiber J., Sonnenburg T., Luecke E. (2020). Inhaler devices in asthma and COPD patients—A prospective cross-sectional study on inhaler preferences and error rates. BMC Pulm. Med..

[57] Kamps A.W., van Ewijk B., Roorda R.J., Brand P.L.P. (2000). Poor inhalation technique, even after inhalation instructions, in children with asthma. Pediatr. Pulmonol..

[58] Almomani B.A., Al-Qawasmeh B.S., Al-Shatnawi S.F., Awad S., Alzoubi S.A. (2021). Predictors of proper inhaler technique and asthma control in pediatric patients with asthma. Pediatr. Pulmonol..

[59] Volerman A., Balachandran U., Zhu M., Akel M., Hull A., Siros M., Luna V., Xu I., Press V.G. (2023). Evaluating inhaler education interventions for hospitalized children with asthma: A randomized controlled trial. Ann. Allergy Asthma Immunol..

[60] Capanoglu M., Dibek Misirlioglu E., Toyran M., Civelek E., Kocabas C.N. (2015). Evaluation of inhaler technique, adherence to therapy and their effect on disease control among children with asthma using metered dose or dry powder inhalers. J. Asthma.

[61] Chrystyn H., van der Palen J., Sharma R., Barnes N., Delafont B., Mahajan A., Thomas M. (2017). Device errors in asthma and COPD: Systematic literature review and meta-analysis. npj Prim. Care Respir. Med..

[62] Calzetta L., Aiello M., Frizzelli A., Ritondo B.L., Pistocchini E., Rogliani P., Chetta A. (2022). Impact of Sex on Proper Use of Inhaler Devices in Asthma and COPD: A Systematic Review and Meta-Analysis. Pharmaceutics.

[63] Ocakli B., Ozmen I., Tuncay E.A., Gungor S., Ozalp A., Yasin Y., Adiguzel N., Gungor G., Karakurt Z. (2020). Influence of Gender on Inhaler Technique. Respir. Care.

[64] Wieshammer S., Dreyhaupt J. (2008). Dry powder inhalers: Which factors determine the frequency of handling errors?. Respiration.

[65] Barnestein-Fonseca P., Cotta-Luque V.M., Aguiar-Leiva V.P., Leiva-Fernández J., Martos-Crespo F., Leiva-Fernández F. (2022). The importance of reminders and patient preferences to improve inhaler technique in older adults with COPD. Front. Pharmacol..

[66] Press V.G., Arora V.M., Kelly C.A., Carey K.A., White S.R., Wan W. (2020). Effectiveness of Virtual vs In-Person Inhaler Education for Hospitalized Patients with Obstructive Lung Disease: A Randomized Clinical Trial. JAMA Netw. Open.

[67] Al-Kharouf M.S., Abdeljalil M.H., Obeidat N.M., Al Oweidat K., Awwad O., Rababa M.J. (2023). Video-based teach-to-goal intervention on inhaler technique on adults with asthma and COPD: A randomized controlled trial. PLoS ONE.

[68] Imanipour M., Molazem Z., Rakhshan M., Fallahi M.J. (2022). Evaluation of the Effectiveness of Teach-back Training on Asthma Control Indicators. Tanaffos.

[69] Abbas M.A., Tariq O., Bin Zafar S., Jamil M.I., Hamid K., Iqbal A., Ahmed A., Naeem I. (2024). Improvement in Inhaler Techniques After Training and Counseling in Patients with Chronic Obstructive Pulmonary Disease or Asthma. Cureus.

[70] Sadowski C.A., Cor K., Cave A., Banh H.L. (2015). Administration Technique and Acceptance of Inhaler Devices in Patients with Asthma or COPD. Ann. Pharmacother..

[71] Lenney J., Innes J.A., Crompton G.K. (2000). Inappropriate inhaler use: Assessment of use and patient preference of seven inhalation devices. Respir. Med..

[72] Miszczuk M., Domagała I., Dąbrowska M., Maskey-Warzęchowska M., Krenke R. (2020). How to assess inhalation technique in patients with asthma and COPD—Comparison of three different methods. Eur. Respir. J..

