# Supplementary material for: Expectations Versus Reality in Inhalation Technique—A Case–Control Study of Inhalation Technique in Patients with Asthma or COPD
Source: J Clin Med. 2025 Sep 27;14(19):6848. doi: 10.3390/jcm14196848 (PMC12525108; doi:10.3390/jcm14196848)
Supplement: Supplementary file 1 [file jcm-14-06848-s001.zip › jcm-3870289-supplementary/Dabrowska Supplemental Data CleanVersion19.09.25.pdf]

Supplementary Table S1. Checklist of the potential mistakes during inhalation

	Type of mistakes	Critical mistakes
**MDI/ SMI**		
1	No cap removal	x
2	No shaking the inhaler before use	
3	No exhalation before use	
4	Incorrect position of inhaler during inhalation	x
5	Actuation against closed lips, teeth	x
6	More than one actuation for a single inhalation	x
7	Actuation in the second half of inspiration	x
8	Actuation after the inspiration	x
9	Too rapid and forceful inhalation	x
10	Too early stopping of inhalation	
11	Inhalation through nose while actuation	
12	No or too short breath holding after inhalation	
13	Not sealing lips around mouthpiece during inhalation	
**DPI**		
1	Failure to open the device	x
2	Not loading capsule	x
3	Not piercing capsule	x
4	Not loading dose before inhaling	x
5	Breathing out into inhaler	
6	Inhaling through the nose	
7	Premature termination of inhalation	
8	Not sealing lips around mouthpiece during inhalation	x
9	Slow and not forceful inspiration	x
10	No or too short breath holding after inhalation	
11	No repeated inhalation from the same capsule	
12	No control of drug powder remnants after drug intake	
13	No exhalation before use	
Abbreviations: DPI, Dry Powder Inhaler; MDI, Meter Dose Inhaler; SMI, Soft Mist Inhaler		

Supplementary Table S2. Questionnaire assessing motivation for treatment

**Questionnaire: Motivation for Treatment**		
	YES	NO
In your opinion, does your lung disease require medical treatment?		
Do you take your inhaled medications regularly as prescribed by your physician?		
Do you find the use of inhalers for medication administration to be easy?		
Do you consider yourself capable of correctly administering inhaled medication?		

Do you believe that inhaled medications are effective in helping your condition?		
Are you currently using any alternative therapies for asthma or COPD management?		
During the past six months, have you experienced any discontinuation of your inhaled therapy for more than one day?		
Please rate your motivation to follow treatment on a scale from 0 (no motivation) to 10 (maximal motivation)		

Supplementary Table S3. Factors that impact on proper inhalation technique- univariate analysis

**Factor**	**Odds Ratio**	**Standard error**	**P value**
Significant visual disorders	0.399	0.369	0.013
Presence of atopy	2.308	0.409	0.041
Good self-assessment of inhalation technique	6.968	0.806	0.016
Deviations from regular use of inhaler during last 6 months	0.457	0.381	0.040
Presence of side effects related to inhalers	2.512	0.460	0.045
Use of MDI	0.319	0.428	0.008
